# Paracrine effects of human adipose-derived mesenchymal stem cells in inflammatory stress-induced senescence features of osteoarthritic chondrocytes

**DOI:** 10.18632/aging.101007

**Published:** 2016-08-02

**Authors:** Julia Platas, Maria Isabel Guillén, Maria Dolores Pérez del Caz, Francisco Gomar, Miguel Angel Castejón, Vicente Mirabet, Maria José Alcaraz

**Affiliations:** ^1^ Department of Pharmacology and IDM, University of Valencia, Burjasot, 46100 Valencia, Spain; ^2^ Department of Pharmacy, Cardenal Herrera-CEU University, Moncada, 46113 Valencia, Spain; ^3^ Department of Burn and Plastic Surgery, La Fe Polytechnic University Hospital, 46026 Valencia, Spain; ^4^ Department of Surgery, Faculty of Medicine, University of Valencia, 46010 Valencia, Spain; ^5^ Department of Orthopaedic Surgery and Traumatology, De la Ribera University Hospital, Alzira, 46600 Valencia, Spain; ^6^ Valencia Transfusion Center, Generalitat Valenciana, 46014 Valencia, Spain

**Keywords:** adipose-derived mesenchymal stem cells conditioned medium, inflammation, senescence, chondrocytes

## Abstract

Aging and exposure to stress would determine the chondrocyte phenotype in osteoarthritis (OA). In particular, chronic inflammation may contribute to stress-induced senescence of chondrocytes and cartilage degeneration during OA progression. Recent studies have shown that adipose-derived mesenchymal stem cells exert paracrine effects protecting against degenerative changes in chondrocytes. We have investigated whether the conditioned medium (CM) from adipose-derived mesenchymal stem cells may regulate senescence features induced by inflammatory stress in OA chondrocytes. Our results indicate that CM down-regulated senescence markers induced by interleukin-1β including senescence-associated β-galactosidase activity, accumulation of γH2AX foci and morphological changes with enhanced formation of actin stress fibers. Treatment of chondrocytes with CM also decreased the production of oxidative stress, the activation of mitogen-activated protein kinases, and the expression of caveolin-1 and p21. The effects of CM were related to the reduction in p53 acetylation which would be dependent on the enhancement of Sirtuin 1 expression. Therefore, CM may exert protective effects in degenerative joint conditions by countering the premature senescence of OA chondrocytes induced by inflammatory stress.

## INTRODUCTION

Osteoarthritis (OA) is the most common joint disorder affecting aging people [1]. The OA chondrocyte phenotype could be the result of aging and exposure to stresses such as mechanical loading, oxidative stress and inflammation. Therefore, chronic production of inflammatory mediators may play an important role in articular degradation [2, 3]. Senescence markers have been detected in cartilage from OA patients and it is believed that chondrocyte senescence contributes to the age-related increase in the prevalence of OA and reduced efficacy of cartilage repair. In late OA, failure of repair responses due to cell senescence would result in a progressive degeneration of cartilage [4]. As chondrocytes do not normally proliferate in the articular cartilage of adults [5], chondrocyte senescence seems unlikely to result from multiple cycles of cell proliferation and repetitive stress may be a main cause [6]. In addition to the natural senescence of aging, exposure to pro-inflammatory and oxidative mediators has been implicated in stress-induced premature senescence [7]. In particular, pro-inflammatory cytokines such as interleukin(IL)-1β and tumor necrosis factor α could contribute to an imbalance between anabolic and degradative mechanisms which may result in extrinsic stress-induced senescence of articular chondrocytes [8].

The type III histone/protein deacetylase Sirt1 exerts diverse physiological functions mainly mediated by deacetylation of histones, transcription factors or coactivators such as p53, forkhead box O (FOXO), peroxisome proliferator-activated receptor γ, etc. Sirt1 has been shown to regulate stress resistance, inflammation and senescence (reviewed in [9]). In chondrocytes, Sirt1 appears to play a protective role. Studies in human cartilage have suggested that Sirt1 is involved in the pathogenesis of OA through the modulation of gene expression. Therefore, Sirt1 may regulate the survival of chondrocytes [10] and the expression of cartilage-specific genes [11] besides the inhibition of hypertrophy [12] and senescence [13].

Mesenchymal stem cells appear to emerge as a promising therapy in many types of tissue/organ injuries. These cells release a number of factors that promote angiogenesis, immunomodulation and recruitment of stem/progenitor cells followed by cell differentiation, proliferation and synthesis of extracellular matrix [14]. A wide range of evidence has demonstrated the interest of adipose-derived mesenchymal stem cells (AMSC) in tissue regeneration and immunomodulation. As the pharmacological treatment of OA does not modify the structural changes associated with disease, novel approaches such as injection of autologous and allogeneic stem cells derived from various sources (e.g. bone marrow, adipose tissue, etc.) or differentiation into cartilage using scaffolds have been explored [14, 15]. In the context of cartilage protection, administration of AMSC into the knee joint during the early stage of experimental OA inhibited synovial activation and prevented cartilage damage [16, 17].

A number of studies have demonstrated the role of soluble factors produced by stem cells as mediators of their therapeutic effects [15, 18, 19]. These factors may contribute to the inhibition by AMSC of degenerative changes in a rabbit OA model [20]. In this regard, paracrine effects appear to be responsible for the anti-inflammatory [21, 22] and anti-fibrotic [23] properties of AMSC in human OA chondrocytes. However, little is known of senescence regulation by AMSC in human OA chondrocytes. In the present study we have investigated whether human AMSC conditioned medium (CM) may modify inflammatory stress-induced senescence features of OA chondrocytes.

## RESULTS

### CM decreases the number of senescent cells

**Figure 1:**
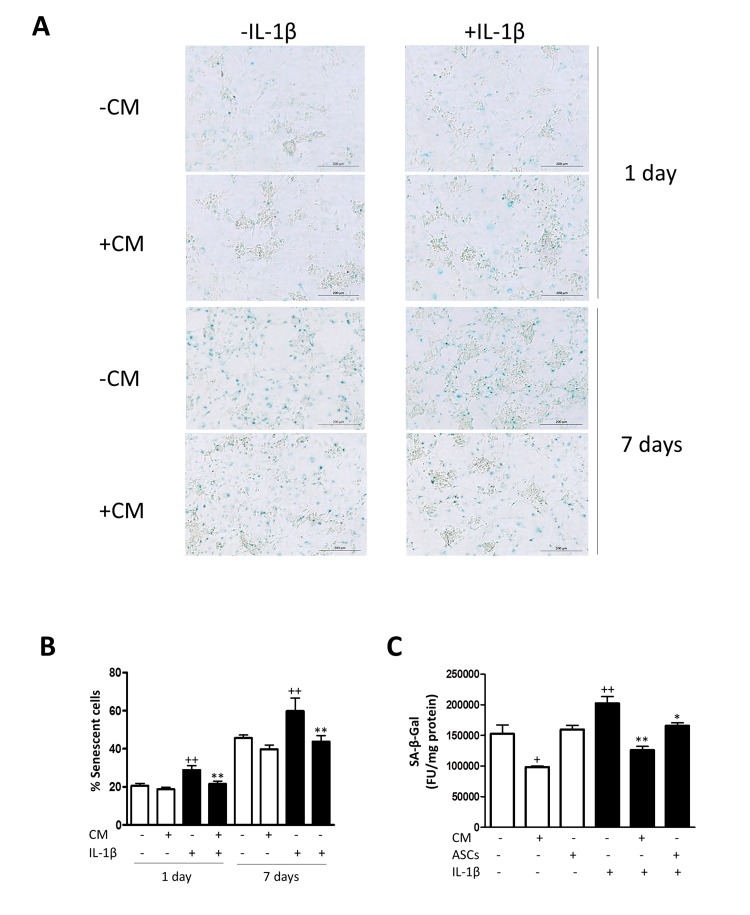
Induction of SA-β-Gal by IL-1β in OA chondrocytes (**A**) Representative light microscopy images of cells stained with the senescent marker SA-β-Gal in monolayer cultures of OA chondrocytes. Bars: 200 μm. (**B**) Percentage of SA-β-Gal positive cells. OA chondrocytes were incubated with IL-1β and/or CM for 1 day and 7 days. (**C**) SA-β-Gal was quantified by fluorometry. A co-culture system of AMSC and OA chondrocytes was used. Data are shown as mean±standard deviation of N=4 separate experiments with cells from separate donors. +p<0.05, ++p<0.01 with respect to non-treated cells; *p<0.05, **p<0.01 with respect to IL-1β-treated cells. FU: fluorescence units.

In order to characterize the effects of CM on senescence features of OA chondrocytes, we first assessed the marker senescence-associated β-galactosidase (SA-β-Gal). In primary chondrocytes, we observed that IL-1β induced a significant increase in the percentage of cells positive for SA-β-Gal at days 1 and 7 compared with non-stimulated cells (Fig. 1A and B). At both time points, CM treatment resulted in a significant reduction in the percentage of SA-β-Gal positive chondrocytes in the presence of IL-1β stimulation. The levels of SA-β-Gal staining became elevated at day 7 with respect to day 1 in all groups, which may be related to the higher cell density at day 7, as reported for human fibroblastic cells [24]. In addition, we studied the marker SA-β-Gal in a co-culture system of AMSC and OA chondrocytes and results were quantified by fluorometry in a micro-plate reader. As shown in Fig. 1C, the presence of AMSC significantly reduced the level of SA-β-Gal of OA chondrocytes induced by IL-1β stimulation. This assay confirmed the effect of CM on this marker which was significantly reduced even in the absence of IL-1β.

### CM inhibits the accumulation of γH2AX foci

**Figure 2:**
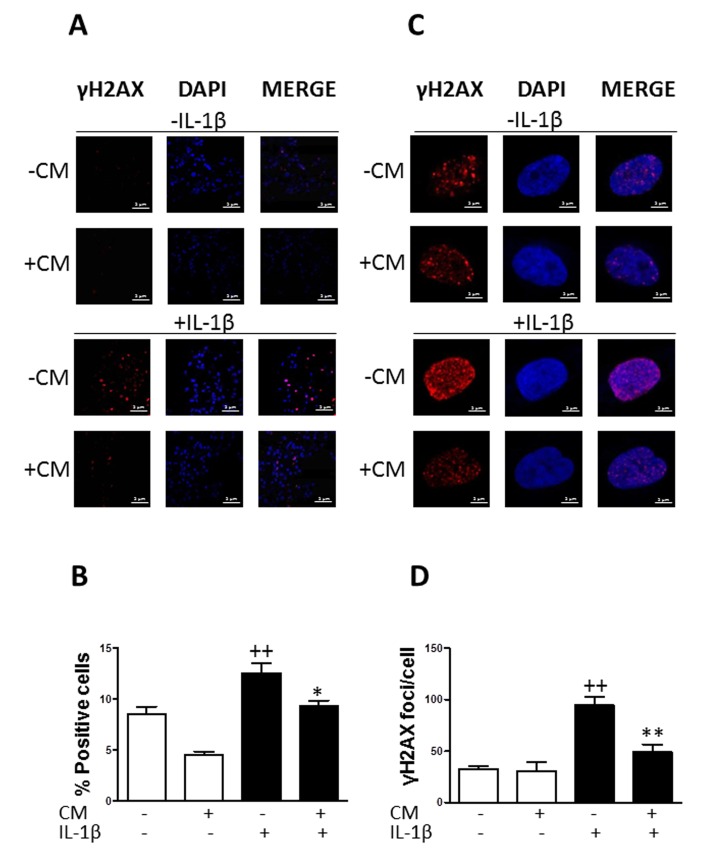
Effect of CM on γ-H2AX foci accumulation induced by IL-1β in OA chondrocytes (**A**) Presence of γH2AX (red pixels) positive cells in a representative experiment. DAPI was used to stain the nuclei. Bars: 50μm. (**B**) Percentage of positive cells. (**C**) Presence of γH2AX (red pixels) foci in cell nucleus in a representative experiment. DAPI was used to stain the nuclei. Bars: 5μm. (**D**) Number of γH2AX foci per cell. OA chondrocytes were incubated with IL-1β and/or CM for 3 days. Data are shown as mean±standard deviation of N=4 separate experiments with cells from separate donors. ++p<0.01 with respect to non-treated cells; *p<0.05, **p<0.01, with respect to IL-1β-treated cells.

The presence of γH2AX foci correlates with age and chronic conditions connected with the accumulation of genome damage [25]. Immunofluorescence analyses showed a significant increase in the phosphorylation of the histone variant H2AX (γH2AX) in cells treated with IL-1β with respect to non-stimulated chondrocytes. Approximately 13 % of cells positive for γH2AX (Fig. 2A and B) and 100 foci per nucleus (Fig. 2C and D) were detected in the presence of IL-1β after 3 days of incubation. At earlier times we did not observe any significant changes in the presence of this cytokine (data now shown). We investigated the effects of CM treatment and observed a significant reduction in the percentage of cells positive for γH2AX (of 30%) as well as in the number of γH2AX foci per nucleus (of 50%).

### Effects of CM on actin stress fibers

**Figure 3:**
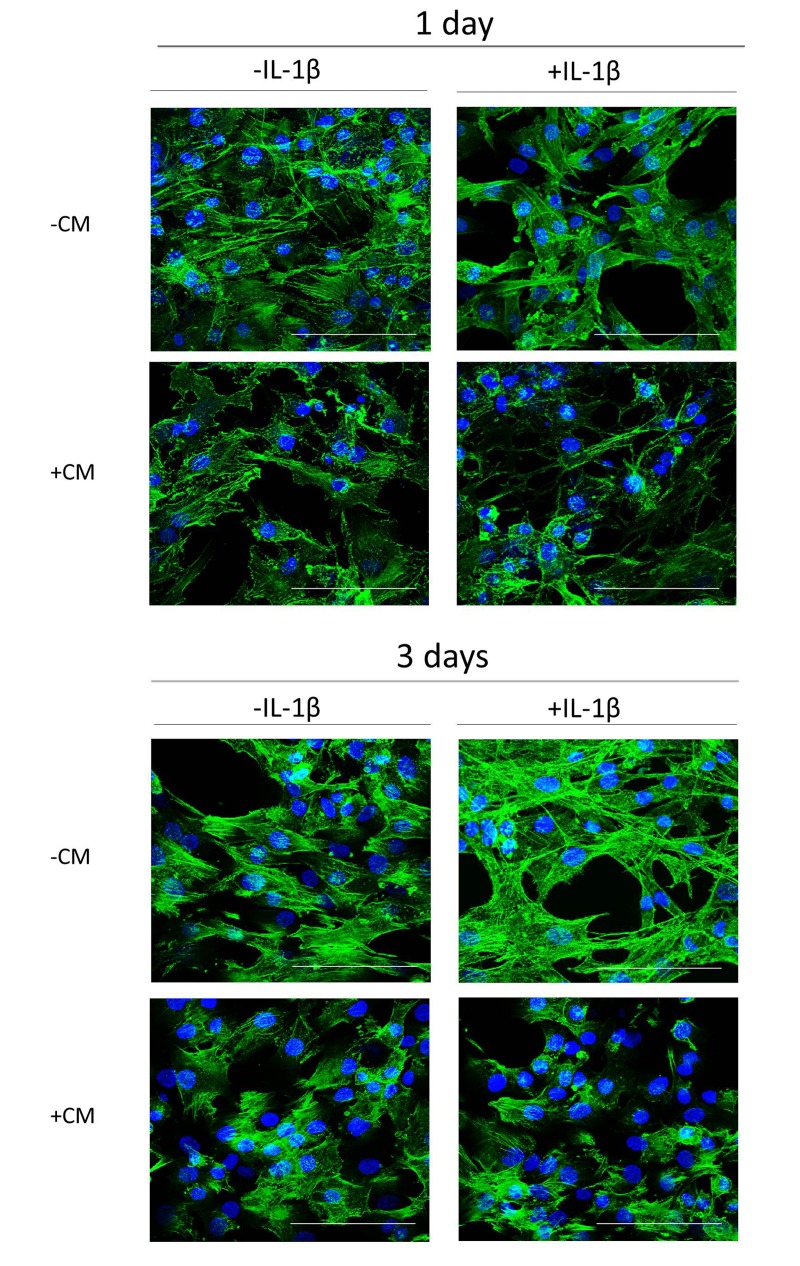
Actin stress fibers formation induced by IL-1β in OA chondrocytes and effect of CM Presence of actin stress fibers (green pixels) in a representative experiment of N=4 separate experiments with cells from separate donors. DAPI was used to stain the nuclei. Bars: 50 μm. OA chondrocytes were incubated with IL-1β and/or CM for 1 day and 3 days.

Morphologic changes are characteristic of the senescent phenotype. Senescent cells show flattened and enlarged shapes with enhancement of actin stress fibers. Fig. 3 shows that IL-1β stimulation of OA chondrocytes resulted in cell enlargement with increased actin stress fibers formation after 3 days of incubation. In contrast, cells treated with CM tended to keep their normal morphology and exhibited less actin fibers formation.

### Effects of CM on oxidative stress

**Figure 4:**
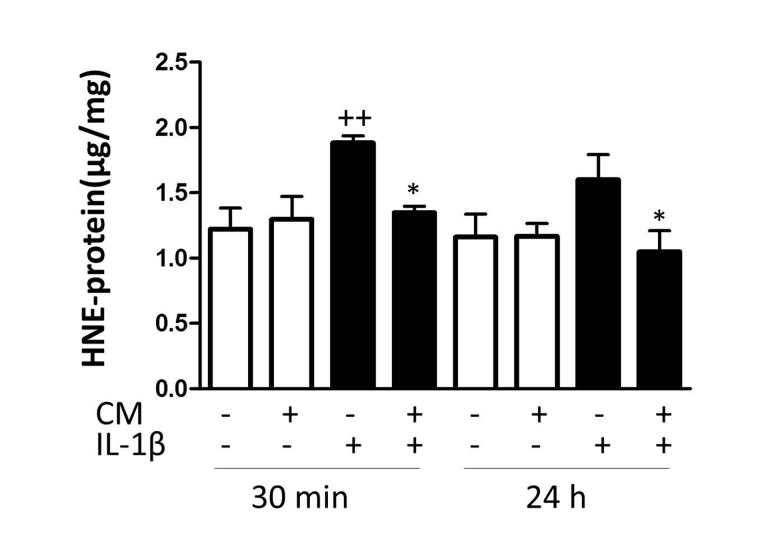
Effect of CM on oxidative stress induced by IL-1β in OA chondrocytes The intracellular levels of HNE-modified proteins were quantified by ELISA. OA chondrocytes were incubated with IL-1β and/or CM for the indicated times. Data are shown as mean±standard deviation of N=9 separate experiments with cells from separate donors. ++p<0.01 with respect to non-treated cells; *p<0.05, with respect to IL-1β-treated cells.

In human chondrocytes, oxidative stress may lead to DNA damage and senescence [26]. In addition, reactive oxygen species (ROS) production plays an important role in signaling pathways activated by IL-1β in human chondrocytes [27]. As oxidative stress is a key process in the induction and maintenance of senescence, we next investigated the effects of CM on protein modification by ROS. In OA chondrocytes, IL-1β quickly induced the production of ROS and enhanced levels of 4-hydroxy-2-nonenal (HNE)-modified proteins were detected (Fig. 4) whereas CM significantly reduced the amount of HNE-modified proteins.

### Effects of CM on mitogen-activated protein kinases (MAPK)

**Figure 5:**
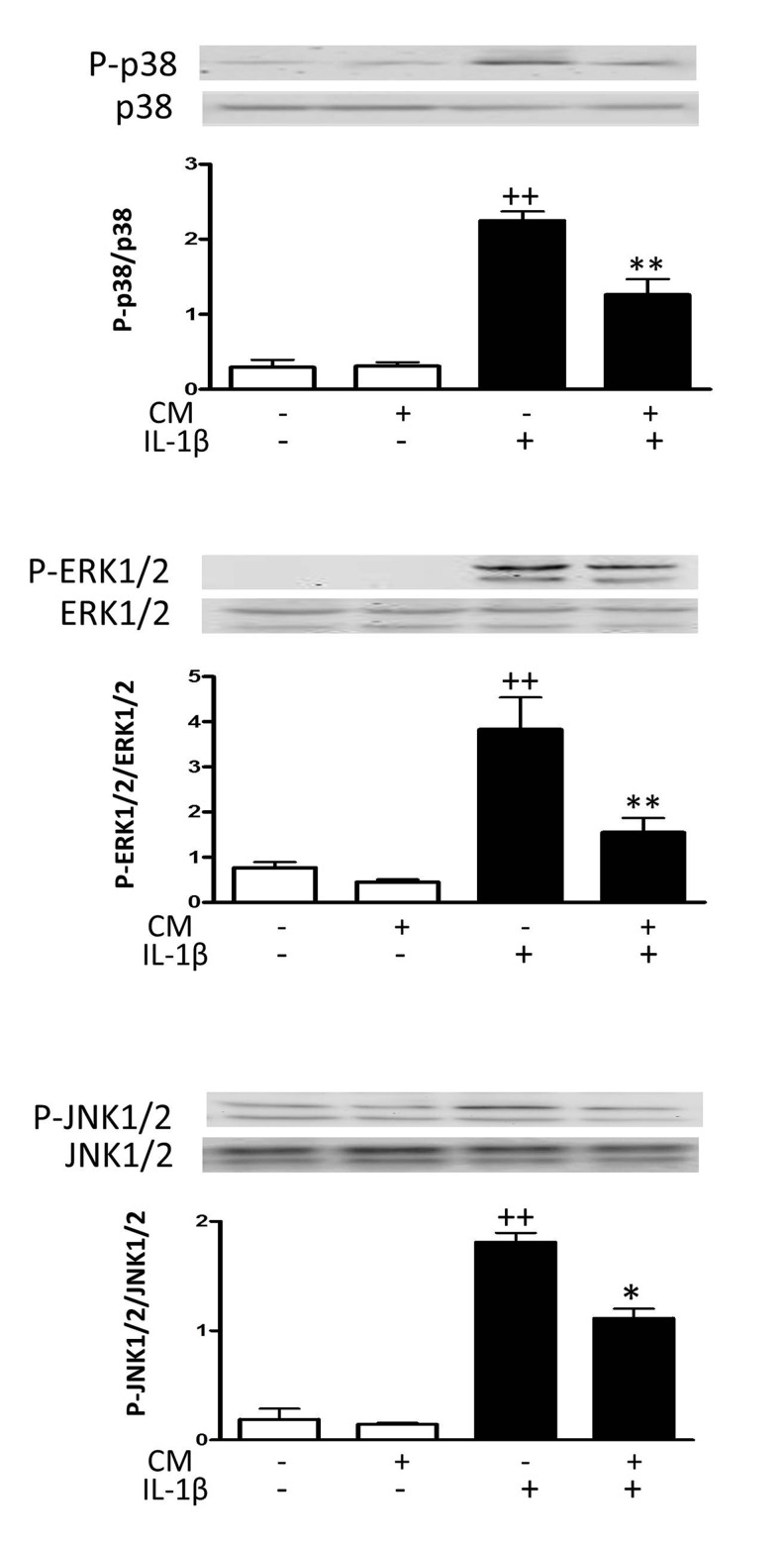
Effect of CM on MAPK OA chondrocytes were incubated with IL-1β and/or CM for 15 min and immunoblotting was performed for phosphorylated and total p38, ERK1/2 and JNK1/2. Data are shown as mean±standard deviation of N=3 separate experiments with cells from separate donors. ++p<0.01 with respect to non-treated cells; *p<0.05, **p<0.01 with respect to IL-1β-treated cells.

We next assessed the activation of signaling proteins that are involved in the oxidative stress response pathway. Fig. 5 shows that IL-1β quickly induced the phosphorylation of mitogen-activated protein kinases (MAPK) extracellular signal-regulated kinase 1/2 (ERK1/2), p38 and c-jun N-terminal kinase 1/2 (JNK1/2). Treatment of OA chondrocytes with CM significantly reduced the phosphorylation of ERK1/2 and p38 and to a lower extent the phosphorylation of JNK1/2.

### Effects of CM on the expression of senescence-associated mediators

To elucidate the mechanisms of CM protective effects against senescence induced by IL-1β, we examined whether CM regulates the expression of key senescence-associated mediators. We used enzyme-linked immunosorbent assay (ELISA) and western blotting to assess the protein levels of caveolin-1, p53 and cyclin-dependent kinase inhibitors p21 and p16.

**Figure 6:**
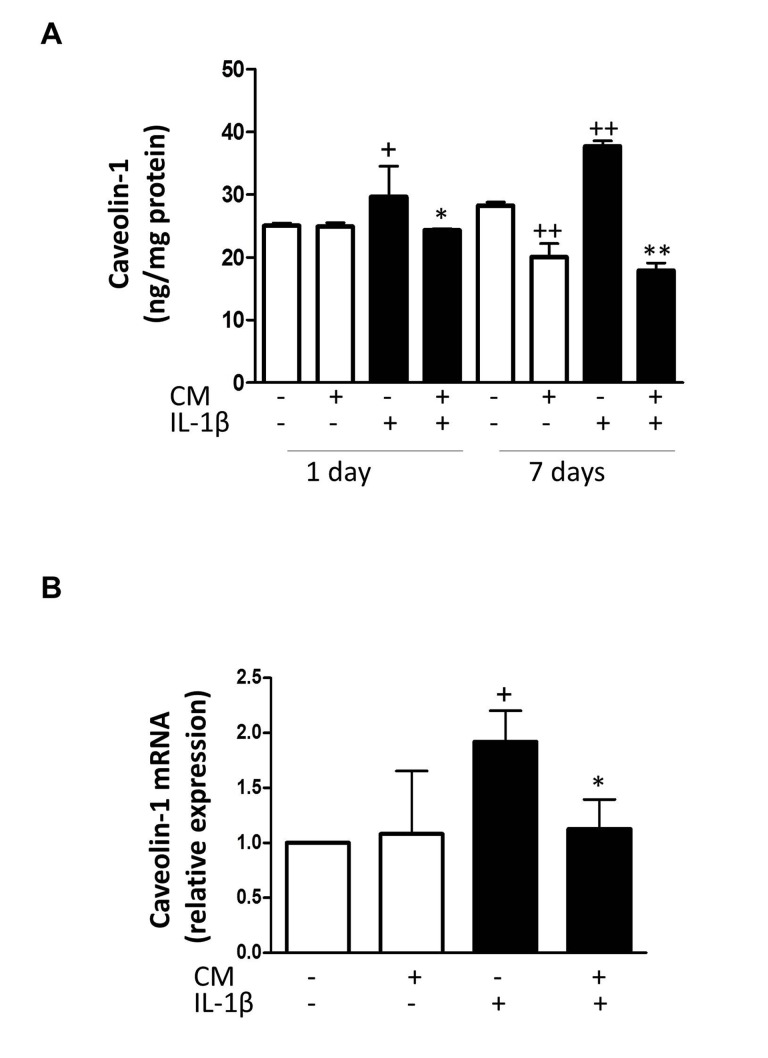
Effect of CM on caveolin-1 (**A**) Protein levels of caveolin-1 were assessed by ELISA. OA chondrocytes were incubated with IL-1β and/or CM for the indicated times. (**B**) Caveolin-1 mRNA expression was determined by quantitative real-time PCR. OA chondrocytes were incubated with IL-1β and/or CM for 24 hours. Data are shown as mean±standard deviation of N=4 separate experiments with cells from separate donors. +p<0.05, ++p<0.01 with respect to non-treated cells; *p<0.05, **p<0.01 with respect to IL-1β-treated cells.

**Figure 7:**
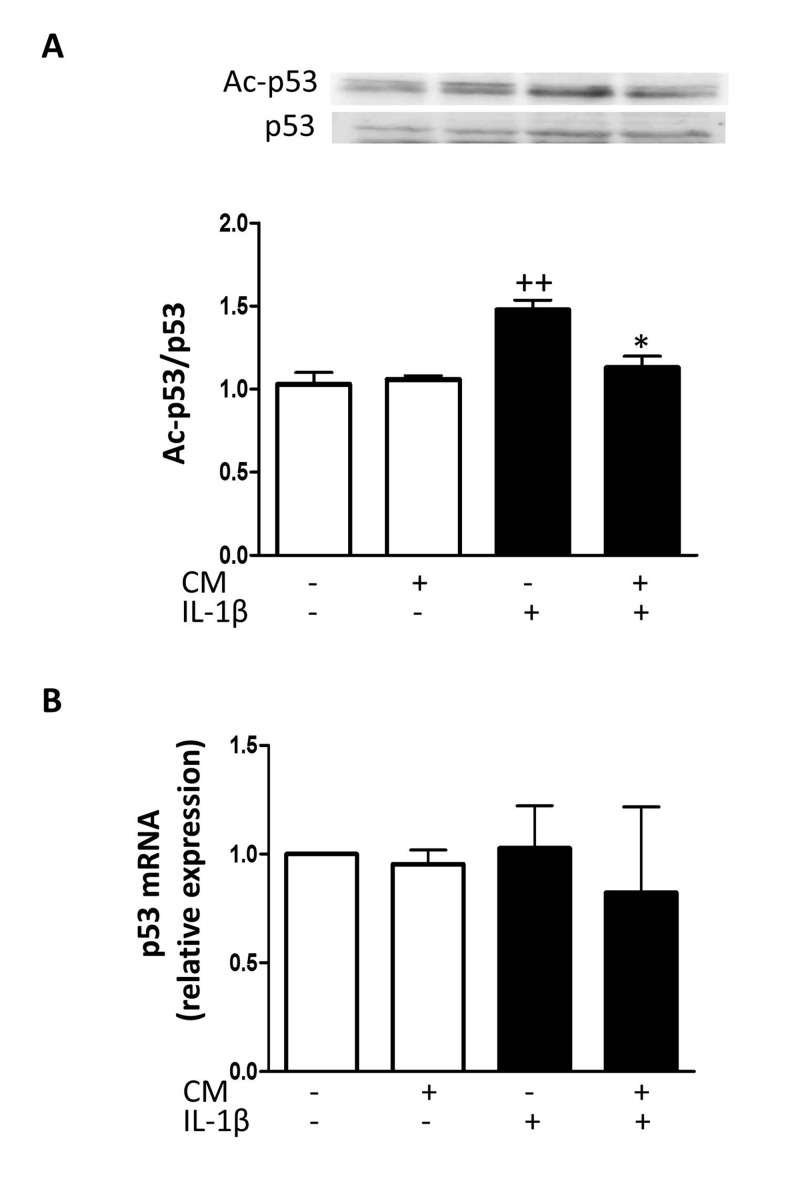
Effect of CM on p53 (**A**) Immunoblotting was performed for acetylated and total p53. (**B**) p53 mRNA expression was determined by quantitative real-time PCR. OA chondrocytes were incubated with IL-1β and/or CM for 24 hours. Data are shown as mean±standard deviation of N=4 separate experiments with cells from separate donors. ++p<0.01 with respect to non-treated cells; *p<0.05 with respect to IL-1β-treated cells.

**Figure 8:**
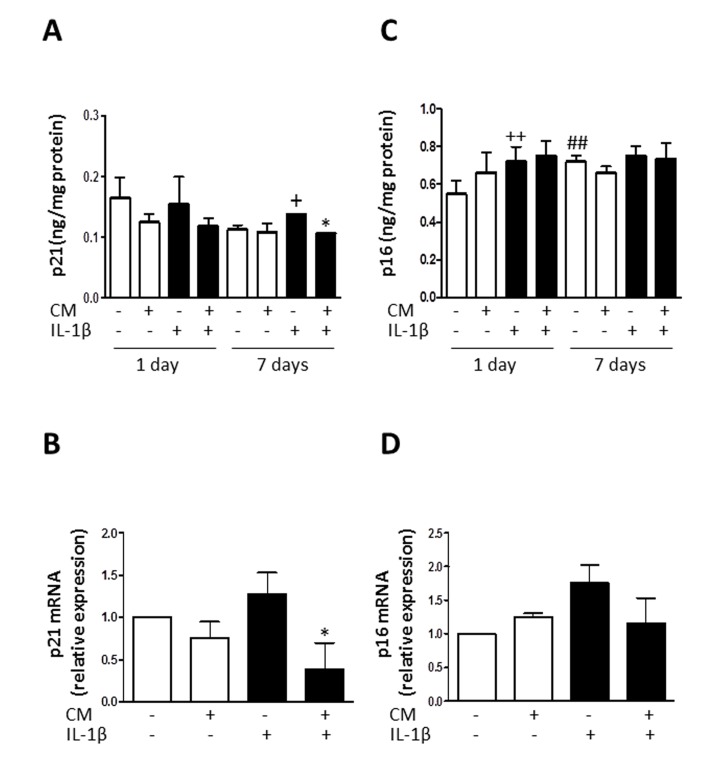
Effect of CM on p21 and p16 (**A**) Protein levels of p21 were assessed by ELISA. OA chondrocytes were incubated with IL-1β and/or CM for the indicated times. (**B**) p21 mRNA expression was determined by quantitative real-time PCR. OA chondrocytes were incubated with IL-1β and/or CM for 24 hours. (**C**) Protein levels of p16 were assessed by ELISA. OA chondrocytes were incubated with IL-1β and/or CM for the indicated times. (**D**) p16 mRNA expression was determined by quantitative real-time PCR. OA chondrocytes were incubated with IL-1β and/or CM for 24 hours. Data are shown as mean±standard deviation of N=8 separate experiments with cells from separate donors. +p<0.05, ++p<0.01 with respect to non-treated cells; *p<0.05 with respect to IL-1β-treated cells; ##p<0.01 with respect to non-treated cells at day 1.

mRNA expression was determined by quantitative RT-PCR and expression rates relative to controls of non-stimulated chondrocytes from the same patients were determined. Caveolin-1 is a mediator of stress-induced cellular senescence, such as premature senescence induced by oxidative stress and IL-1β in articular chondrocytes [28]. IL-1β treatment of OA chondrocytes enhanced the expression of caveolin-1 both at protein and mRNA levels (Fig. 6) whereas CM counteracted this induced expression. In addition, IL-1β increased the acetylation of p53 without changes in protein and mRNA (Fig. 7). CM treatment of OA chondrocytes significantly reduced p53 acetylation. Our results thus indicate that IL-1β induces the activation of p53 and that CM is able to decrease this response. p53 activation is known to result in the induction of p21^WAF1/Cip1^. Thus, we next assessed p21 protein and mRNA expression in OA chondrocytes stimulated with IL-1β either in the presence or absence of CM. Significant increases in p21 protein were detected at day 7 of IL-1β incubation (Fig. 8A) and treatment with CM resulted in the down-regulation of p21 protein and mRNA levels (Fig. 8A and B). IL-1β induced a significant increase in p16 protein at day 1 (Fig. 8C and D). In non-stimulated cells, the levels of p16 protein were enhanced at day 7 compared with day 1 and they did not increase further in the presence of IL-1β. Treatment with CM did not modify p16 mRNA or protein levels.

### Effects of CM on sirtuin 1 (Sirt1)

**Figure 9:**
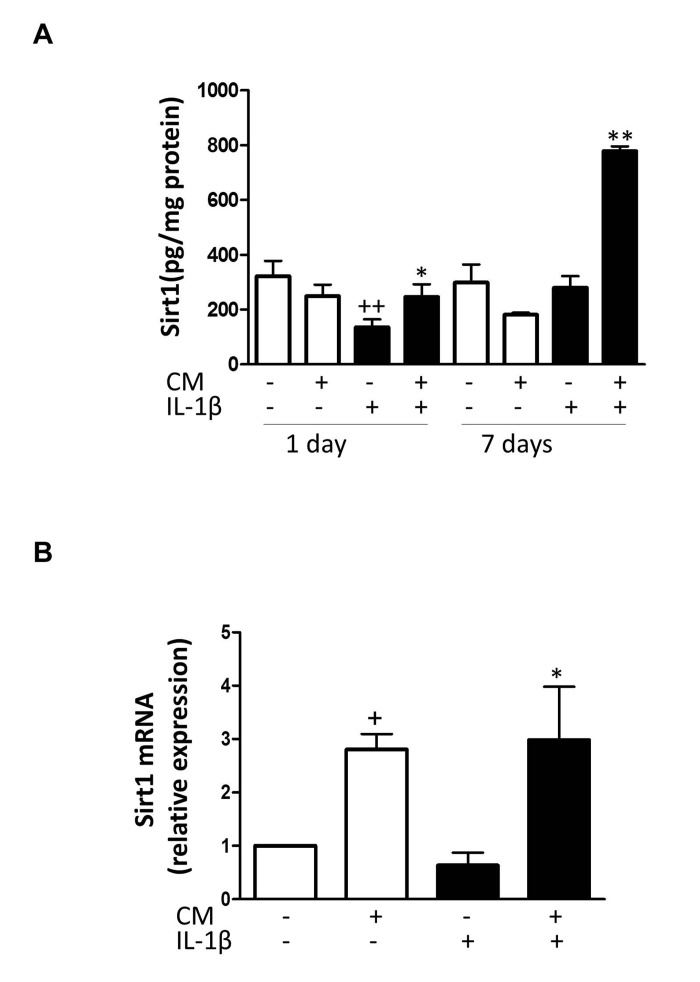
Effect of CM on Sirt1 (**A**) Protein levels of Sirt1 were assessed by ELISA. OA chondrocytes were incubated with IL-1β and/or CM for the indicated times. (**B**) Sirt1 mRNA expression was determined by quantitative real-time PCR. OA chondrocytes were incubated with IL-1β and/or CM for 24 hours. Data are shown as mean±standard deviation of N=8 separate experiments with cells from separate donors. +p<0.05, ++p<0.01 with respect to non-treated cells; *p<0.05, **p<0.01 with respect to IL-1β-treated cells.

**Figure 10:**
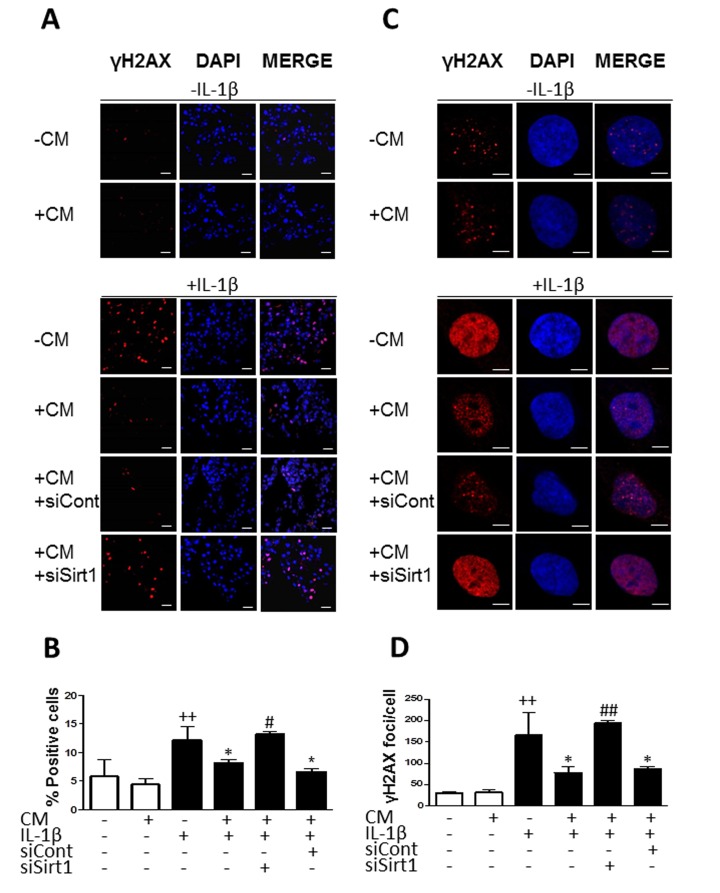
Effect of Sirt1 siRNA on the reduction of γH2AX foci accumulation by CM (**A**) Representative experiment showing that Sirt1 siRNA (siSirt1) inhibits the effect of CM on the accumulation of γH2AX (red pixels) positive cells induced by IL-1β. siCont: non-specific siRNA. DAPI was used to stain the nuclei. Bars: 50μm. (**B**) Percentage of positive cells. (**C**) Representative experiment showing that Sirt1 siRNA (siSirt1) inhibits the effect of CM on the accumulation of γH2AX (red pixels) foci per cell induced by IL-1β. siCont: non-specific siRNA. DAPI was used to stain the nuclei. Bars: 5μm. (**D**) Number of γH2AX foci per cell. OA chondrocytes were incubated with the indicated treatments for 3 days. Data are shown as mean±standard deviation of N=4 separate experiments with cells from separate donors. ++p<0.01 with respect to non-treated cells; *p<0.05 with respect to IL-1β-treated cells; #p<0.05, ##p<0.01 with respect to IL-1β+CM.

To better understand how CM regulates stress-induced senescence in OA chondrocytes, we next investigated the contribution of sirtuin 1 (Sirt1). Fig. 9A shows that IL-1β reduced the expression of Sirt1 protein at day 1 whereas CM enhanced it with a strong effect at day 7. In addition, CM significantly increased mRNA expression of Sirt1 (Fig. 9B). To confirm the involvement of Sirt1 in CM effects on IL-1β-induced senescence, Sirt1 was knocked down by using a specific siRNA. We then compared the effects of CM on γH2AX accumulation induced by IL-1β at day 3 in cells transfected with Sirt1 siRNA, cells transfected with a non-targeting siRNA control and non-transfected cells. As shown in Fig. 10, Sirt1 knocked down significantly abolished the protective effect of CM on γH2AX accumulation measured either as the percentage of positive cells (Fig. 10A and B) or the number of γH2AX foci per nucleus (Fig. 10C and D).

## DISCUSSION

It is widely thought that inflammation is involved in the pathogenesis of articular cartilage degeneration. Pro-inflammatory cytokines and chemokines induce the production of ROS by articular cells resulting in the activation of key signaling pathways such as MAPK and nuclear factor-κB (NF-κB) [8]. We have shown previously that CM from AMSC inhibits NF-κB activation and the production of inflammatory mediators in OA chondrocytes [21]. In the present study, we have shown that CM down-regulates inflammatory stress-induced senescence in OA chondrocytes.

Cellular senescence is classified into intrinsic telomere-dependent replicative senescence and extrinsic telomere-independent senescence which is induced by several types of stress, such as oxidative stress or pro-inflammatory cytokines [8]. Progressive telomere shortening due to repeated cell division does not explain senescence in quiescent cells such as chondrocytes [29] which would be more related to stress-induced senescence [30]. An important consequence of premature senescence induced by pro-inflammatory cytokines in chondrocytes could be the alteration of homeostasis in the joint decreasing the ability of the cells to maintain and repair the tissue [4]. We have used primary articular chondrocytes to assess the effects of CM on premature senescence induced by IL-1β as passage of articular chondrocytes induces de-differentiation with loss of chondrocyte phenotype. IL-1β induces an inflammatory stress response leading to DNA damage and senescence [31]. Our results indicate that CM protects chondrocytes against metabolic and morphological changes related to senescence which are the consequence of inflammatory stress. Changes in morphology are characteristic features of cell senescence and functional deterioration of senescent cells has been related to these morphological changes [32]. We have shown enlarged OA chondrocytes with a structure of enhanced actin stress fibers after incubation with IL-1β. Interestingly, the accumulation of γH2AX, a marker of inflammation-induced DNA damage, SA-β-Gal and morphological features were significantly counteracted by CM treatment and our results also demonstrated the effects of AMSC on SA-β-Gal.

We have shown that CM down-regulated the production of oxidative stress induced by IL-1β. Oxidative stress could induce telomere dysfunction in chondrocytes with no requirement for cell division, resulting in cartilage aging and the development of OA through a mechanism involving the acceleration of chondrocyte senescence [33]. In addition, ROS may potentiate the effects of mechanical forces and aging in cartilage which results in a pro-inflammatory state and an imbalance of catabolic and anabolic factors [8, 27] leading to cartilage degradation [34-36], inhibition of the synthesis of extracellular matrix molecules and reduced chondrocyte viability [37]. Therefore, the down-regulation of oxidative stress may contribute to the protective effects of CM on chondrocytes [21]. In our model of inflammatory stress by IL-1β, CM reduced the activation of p38 which is involved in the development of senescence in different cell types [38] and in OA chondrocytes stimulated with IL-1β [28]. In addition, CM reduced the phosphorylation of ERK1/2 and to a lesser extent, that of JNK which can also play a role in chondrocyte senescence (reviewed in [39]).

Caveolin-1 is a mediator of stress-induced cellular senescence, such as premature senescence induced by oxidative stress and IL-1β in articular chondrocytes [28]. A wide range of evidence indicates that caveolin-1-rich regions of plasma membrane are associated with receptors and molecules which play an important role in degradative pathways, such as MMPs synthesis and activation [40]. In fact, caveolin-1, β1-integrin and urokinase-type plasminogen activator receptor may form functional complexes in human chondrocytes [41]. Previous reports have demonstrated that caveolin-1 overexpression results in cellular senescence in chondrocytes whereas a reduction in caveolin-1 levels in senescent chondrocytes could modify the aging phenotype [28]. Our data indicate that CM counteracted the increased caveolin-1 expression induced by IL-1β. Caveolin-1 could be involved in morphological changes of senescent cells through the regulation of focal adhesion kinase activity and actin stress fibers formation [32]. Therefore, our results suggest that the reduction in caveolin-1 expression after CM treatment may interfere with the actin stress fibers formation and senescent morphological changes observed in OA chondrocytes stimulated by IL-1β. These data are in line with our previous results showing that CM counteracts the loss of the chondrocyte phenotypic marker type II collagen during inflammatory stress [21].

Caveolin-1 overexpression leads to the up-regulation of the p53/p21 pathway which may mediate stress-induced premature senescence in OA chondrocytes [28]. Our results indicate that CM reduced the activation of this pathway by IL-1β, with a decrease in p53 acetylation and p21 expression. p21 levels increase in senescent articular chondrocytes and at earlier stages in the presence of chronic oxidative stress. In fact, p21 may represent a reliable marker of cumulative DNA damage and senescence in chondrocytes [26]. In addition, p16 may be involved in the processes of chondrocyte aging and OA [42]. Nevertheless, we have shown that p16 levels were not significantly modified by CM treatment, suggesting that this pathway may not be involved in the protective effects of CM on cellular senescence.

It is commonly accepted that Sirt1 is necessary for cartilage homeostasis. In addition to improve chondrocyte survival [43], Sirt1 increases the expression of cartilage-specific genes and modulates NF-κB to repress the synthesis of matrix-degrading enzymes induced by IL-1β [44]. Consequently, the expression of Sirt1 is reduced in OA chondrocytes and subchondral osteoblasts as well as in senescent cells [12, 45]. Consistent with these results, Sirt1 inhibition increases p53 acetylation leading to a premature senescence-like phenotype in different cell types [46] and Sirt1 deficiency in mice results in an increased cartilage degeneration with age and early onset of OA [47].

Sirt1 binds and deacetylates p53 leading to the repression of p53-mediated transactivation and cellular senescence [48]. In addition, Sirt1 regulates oxidative stress and can protect against premature senescence acting through p53, p21, FOXOs and molecules involved in DNA damage and repair (reviewed in [49]). On the other hand, Sirt1 is a redox-sensitive protein subjected to regulation upon different posttranslational modifications. In particular, Sirt1 phosphorylation by JNK1 may be a protective mechanism against the effects of oxidative stress on cell viability [50] although this mechanism may not be involved in the effects of oxidative stress in human articular chondrocytes [13]. As reported in fibroblasts [51], it is likely that caveolin-1 mediates the oxidant-induced inhibition of Sirt1 resulting in p53 acetylation/activation and premature senescence of chondrocytes induced by stress. Another characteristic of senescent cells is the acquisition of a senescence-associated secretory phenotype with release of different factors possessing autocrine and paracrine activities. Our previous findings suggest that CM also down-regulates this response in OA chondrocytes subjected to inflammatory stress as the secretion of IL-6 and MMPs was significantly decreased in the presence of CM [21].

A reduction in Sirt1 induced by oxidant/carbonyl stress may contribute to the pathogenesis of chronic inflammatory conditions (reviewed in [49]). We have shown in OA chondrocytes that IL-1β reduces Sirt1 levels and increases p53 acetylation while treatment with CM protected against these effects of IL-1β and led to the down-regulation of senescence features in OA chondrocytes. In addition, the inhibitory effect of CM on the senescence marker γH2AX was abolished by a Sirt1 siRNA confirming the dependence of this protective effect on Sirt1 activation.

Systemic inflammation in older adults with OA is a factor associated with progression of disease, pain and functional impairment. Excessive mechanical loading of the joint may lead to stress-induced senescence and increased production of pro-inflammatory mediators [52]. Therefore, premature senescence would result in a lower ability of chondrocytes to counteract the many assaults that cartilage suffers with time contributing to the initiation and progression of OA [53]. Stem cells present new opportunities for the development of future therapeutics. Therefore, identifying key AMSC-secreted factors and their functional roles seems a useful approach for rational design of new therapeutical approaches. Although more clinical studies are necessary, a wide range of evidence indicates that intra-articular injection of AMSC, alone or associated to growth factors and/or scaffolds, can improve cartilage quality in OA (reviewed in [54]). These effects of AMSC may be due to the secretion of biological products which could be used as therapeutic agents [18]. AMSC are known to release a variety of cytokines and growth factors (reviewed in [55]). Preliminary experiments have revealed the complex composition of CM including soluble factors and extracellular vesicles (data not shown) that may contribute to the observed effects on chondrocytes. Although further studies are required to know the mechanisms behind the chondroprotective capacity of CM, our data support the interest of AMSC paracrine effects and suggest that CM may have therapeutic potential in the treatment of degenerative joint alterations. In addition to the down-regulation of inflammatory and catabolic mediators [21], CM may exert protective effects by countering the premature senescence of OA chondrocytes induced by inflammatory stress.

## MATERIALS AND METHODS

### Cells and culture media

Adipose tissues were obtained from 9 non-obese donors who had undergone abdominoplasty without any underlying disease (8 women and 1 man, aged 54.1 ± 7.4 years, mean ± SEM). Adipose tissue samples were washed with phosphate-buffered saline (PBS), minced, digested at 37°C for 1 h with 1% of type I collagenase (Gibco, Life Technologies, Madrid, Spain), and filtered through a 100 μm cell strainer (BD Biosciences Durham, NC, USA). The cells were washed with DMEM/HAM F12 containing penicillin and streptomycin (1%), seeded onto tissue culture flasks in DMEM/HAM F12 medium with penicillin and streptomycin (1%) supplemented with 15% human serum, and incubated with 5% CO_2_ at 37°C. Human serum was obtained from whole-blood donations of AB-blood-group-typed donors according to the criteria of Valencia Transfusion Center. At 24 h, when the cells reached the semi-confluence, the tissue culture plates were washed to remove any residual non-adherent cells.

The phenotype of AMSC was analysed by flow cytometry (Flow Cytometer II, BD Biosciences, San Jose, CA, USA) with specific antibodies, anti-CD105-PE, anti-CD90PerCP-eFluo710, anti-CD34APC (eBioscience, Inc., San Diego, CA, USA), and anti-CD45-PE (BD Pharmigen), and cellular viability with propidium iodide. CM was collected from cells at passages 0 and 1 every 48 h of culture, pooled, centrifuged, and stored at −80°C in sterile conditions. The knee cartilage specimens were obtained from patients with the diagnosis of advanced OA (18 women and 6 men, aged 73.2 ± 6.8 years, mean ± SEM) undergoing total knee joint replacement. Diagnosis was based on clinical and radiological evaluation. Cartilage was dissected from the femoral condyles and tibial plateau of the knee joint and diced into small pieces. Human articular chondrocytes were isolated by sequential enzymatic digestion: 1 h with 0.1 mg/ml hyaluronidase (Sigma-Aldrich) followed by 12–15 h with 1 mg/ml collagenase (type IA) (Sigma-Aldrich) in DMEM/HAM F12 (Sigma-Aldrich) containing penicillin and streptomycin (1%) at 37°C in 5% CO_2_ atmosphere. The digested tissue was filtered through a 70 μm nylon mesh (BD Biosciences), washed, and centrifuged. Cell viability was greater than 95% according to the Trypan blue exclusion test. Experiments were performed with chondrocytes in primary culture. Chondrocytes were maintained with 5% CO_2_ at 37°C in DMEM/HAMF12 (Sigma-Aldrich) containing penicillin and streptomycin (1%), supplemented with 10% fetal bovine serum (Sigma-Aldrich).

Cells were incubated for different times in DMEM/HAM F12 (Sigma-Aldrich) containing penicillin and streptomycin (1%) supplemented with 10% human serum, in the presence or absence of IL-1β (10 ng/ml) and/or CM (1 ml of medium for 6-well plates or 2 ml for 3.5 cm plates). In experiments using siRNA (32 nM), transfection was performed in Lipofectamine® RNAiMAX (Invitrogen, Life Technologies S.A., Madrid, Spain), following the manufacturer's recommendations, 24 h before other experimental procedures. Efficiency of knockdown was monitored by quantitative RT-PCR and ELISA. Validated Stealth RNAi^TM^ siRNA for Sirt1 and negative control were from Invitrogen.

The design of the work was approved by the Institutional Ethical Committees (University of Valencia and La Fe Polytechnic University Hospital, Valencia, Spain). Samples were obtained from donors after they provided informed consent according to the Helsinki Declaration of 1975, as revised in 2013.

### Senescence-associated β-galactosidase (SA-β-Gal) assay

SA-β-Gal activity was measured using the cellular senescence staining kit from Cell Biolabs (San Diego, CA, USA). Chondrocytes in primary culture were seeded at 20 × 10^3^ cells/well in Lab-tek chambers (Thermo Scientific, Rochester, NY, USA) and incubated with CM in the presence or absence of IL-1β (10 ng/ml) for 1 or 7 days. Cells were fixed with 0.25% glutaraldehyde in PBS for 5 minutes at room temperature and incubated with staining solution at 4°C overnight. Slides were mounted in Prolong Gold antifade reagent with DAPI (Molecular Probes, Invitrogen, Life Technologies) and examined under a microscope (Leica DM IL LED, Solms, Germany). Slides were photographed with a Leica DFC450 Digital Microscope Camera using the Leica Application Suite software. Cells were quantified with the Image J software using the cell counter complement. Additionally, co-culture experiments were performed with chondrocytes seeded on 6 well-plates (300 × 10^3^ cells/well, 2 ml) and AMSC plated on 24 mm diameter transwell permeable supports (50 × 10^3^ cells/insert, 1 ml) (Corning Costar, Corning, NY, USA), in order to allow soluble factors transit between AMSC and chondrocytes. Cells were incubated in the presence or absence of IL-1β (10 ng/ml) for 7 days and SA-β-Gal assay was performed using the 96-well cellular senescence assay kit, fluorometric format (Cell Biolabs) following manufacturer's instructions. Fluorescence was measured in a Victor3 microplate reader (PerkinElmer, Madrid, Spain).

### Immunofluorescence assay for γH2AX

Chondrocytes in primary culture were seeded at 20 × 10^3^ cells/well in Lab-tek chambers (Thermo Scientific) and incubated with CM in the presence or absence of IL-1β (10 ng/ml) for 3 days. Cells were fixed with 4% formaldehyde in PBS for 15 minutes at room temperature and blocked with 5% normal goat serum and 0.3% Triton X-100 in PBS for 60 minutes at room temperature. Chondrocytes were incubated with Phospho–histone H2AX (Ser139) antibody (Cell Signalling Technology Inc., Beverly, MA, USA) overnight at 4°C. Finally, cells were incubated with Alexa Fluor 546 goat anti-rabbit IgG (H+L) (Thermo Fisher Scientific, MA, USA). Slides were mounted in Prolong Gold antifade reagent with DAPI (Molecular Probes, Invitrogen, Life Technologies S.A., Madrid, Spain) and examined under a confocal microscope (Olympus FV1000, Tokyo, Japan).

### Immunofluorescence assay for F-actin

Chondrocytes in primary culture (20 × 10^3^ cells/well) were seeded in Lab-tek chambers (Thermo Scientific), allowed to grow until near confluence, and stimulated with IL-1β (10 ng/ml) or IL-1β+CM for 1 or 3 days. Cells were fixed with 4% formaldehyde in PBS for 10 minutes at room temperature and blocked with 1% BSA in PBS for 20 minutes at room temperature. Cells were incubated with Alexa Fluor Phalloidin 488 (Molecular Probes, Invitrogen, Life Technologies) for 20 minutes at room temperature. Slides were mounted in Prolong Gold antifade reagent with DAPI (Molecular Probes) and examined under a confocal microscope (Olympus FV1000, Tokyo, Japan).

### Western blot analysis

Cells in primary culture (27 × 10^4^ cells/well in 6-well culture plates) were allowed to grow until nearly confluence and then treated with IL-1β (10 ng/ml) or IL-1β+CM for different times. Cells were lysed with buffer (1% Triton X-100, 1% deoxycholic acid, 20 mM NaCl and 25 mM Tris, pH 7.4) and centrifuged at 4°C for 10 minutes at 10000 xg. Protein content was determined by the DC Bio-Rad Laboratories protein reagent (Bio-Rad, Madrid, Spain). Proteins (25 μg) in supernatants were separated by SDS/PAGE (12.5% gel) and transferred on to PVDF membranes. Membranes were blocked with 3% BSA and incubated with specific polyclonal antibodies against p53 (Novus Biologicals, Littleton, CO, USA), acetyl-p53 (ChemiconMillipore Iberica, Madrid, Spain), P-p38 (Promega Corp., Madison, WI, USA), p38, JNK1/2, P-JNK1/2, ERK1/2, P-ERK1/2 (Cell Signalling Technology Inc.) overnight at 4°C. Membranes were incubated with peroxidase-conjugated goat anti-rabbit IgG (Dako, Copenhagen, Denmark); for p53 we used the secondary antibody anti-mouse IgG (Fc-specific)-peroxidase (Sigma-Aldrich). Finally, membranes with the immunoreactive bands were visualized by ECL® (enhanced chemiluminescence; GE Healthcare, Barcelona, Spain) using an AutoChemi image analyser (UVPInc., Upland, CA, USA).

### Enzyme-Linked Immunosorbent Assay (ELISA)

Several proteins were measured using ELISA kits: p21 (Invitrogen, Barcelona, Spain, with sensitivity of <5 pg/ml), p16 (YH Biosearch Laboratory, Sanghai, China, with sensitivity of 2.24 ng/ml), Sirt1 (Abnova, Walnut, CA, USA, with sensitivity of 30 pg/ml), HNE-proteins (Cell biolabs, San Diego, CA, USA, with sensitivity of 1.56 μg/ml), and caveolin-1 (ElabScience, BioNova Cientifica S.L., Madrid, Spain, with sensitivity of 188 pg/ml). Chondrocytes (27 × 10^4^ cells/well in 6-well culture plates) were allowed to grow until nearly confluence and then stimulated with IL-1β (10 ng/ml) or IL-1β+CM for different times. Cells were lysed with buffer (1% Triton X-100, 1% deoxycholic acid, 20 mM NaCl and 25 mM Tris, pH 7.4) for p21 and HNE-proteins, with specific lysis buffer from the kit for Sirt1, or by repeated freeze-thaw cycles for p16 and caveolin-1. Finally, lysates were centrifuged at 4°C for 10 minutes at 10000 xg. Supernatants were harvested, centrifuged, and frozen at −80°C until analysis.

### Real-time PCR

Chondrocytes were seeded (1.5 × 10^6^ cells) in 3.5 cm plates, allowed to grow until nearly confluence and then stimulated with IL-1β (10 ng/ml) or IL-1β+CM for 24 h. Total RNA was extracted using the TriPure reagent (Roche Applied Science, Barcelona, Spain) according to the manufacturer's instructions. Reverse transcription was accomplished on 1 μg of total RNA using random primers (TaqMan reverse transcription reagents, Applied Biosystems, Spain, Madrid). PCR assays were performed in duplicate on an iCycler Real-Time PCR Detection System using SYBR Green PCR Master Mix (Bio-Rad). Primers were synthesized by Eurofins MWG Operon (Ebersberg, Germany) or Invitrogen. For each sample, differences in threshold cycle (ΔCt) values were calculated by correcting the Ct of the gene of interest to the Ct of the reference gene β-actin. Relative gene expression was expressed as 2^−ΔΔCt^ with respect to non-stimulated cells.

### Statistical analysis

The data were analyzed by one-way analysis of variance followed by Bonferroni's test using the GraphPad Prism 5 software (Graph Pad Software, La Jolla, CA, USA). A p-value of less than 0.05 was considered to be significant.
